# Differential Effects of α‑Synuclein Monomers and Seeds on the Material Properties of Tau Condensates

**DOI:** 10.1021/jacs.6c07726

**Published:** 2026-09-23

**Authors:** Bineet Sharma, Jinying Wang, Priscilla Chinchilla Retana, Jean Baum, Zheng Shi

**Affiliations:** Department of Chemistry and Chemical Biology, Rutgers, 198054 The State University of New Jersey, Piscataway, New Jersey 08854, United States

## Abstract

Tau and α-Synuclein (αSyn) frequently coaggregate in various neurodegenerative disorders. Recently, Tau has been shown to form dynamic, liquid-like condensates that can recruit αSyn, and potentially serve as a precursor to pathological crosstalk and aggregation. However, the quantitative impact of αSyn on the material properties of these condensates remains elusive. Here, we measure the viscosity and interfacial tension of Tau condensates and determine how these properties are modulated by αSyn monomers and fibril seeds. We find that while both forms of αSyn partition efficiently into Tau condensates, they exert vastly different effects on the condensate’s material state. The viscosity of Tau condensates remains unchanged in the presence of αSyn monomers at concentrations up to 200 μM, accompanied by a moderate reduction in the condensates’ interfacial tension. In contrast, adding just 5 μM αSyn fibril seeds triggers rapid maturation of Tau condensates, manifested by a nearly 100-fold increase in their steady-state viscosity within 1 h. These findings provide quantitative insights into condensate mechanics, highlighting the unique capacity of αSyn seeds to modulate Tau condensate viscosity, which may underlie the formation of pathological aggregates.

## Graphical abstract

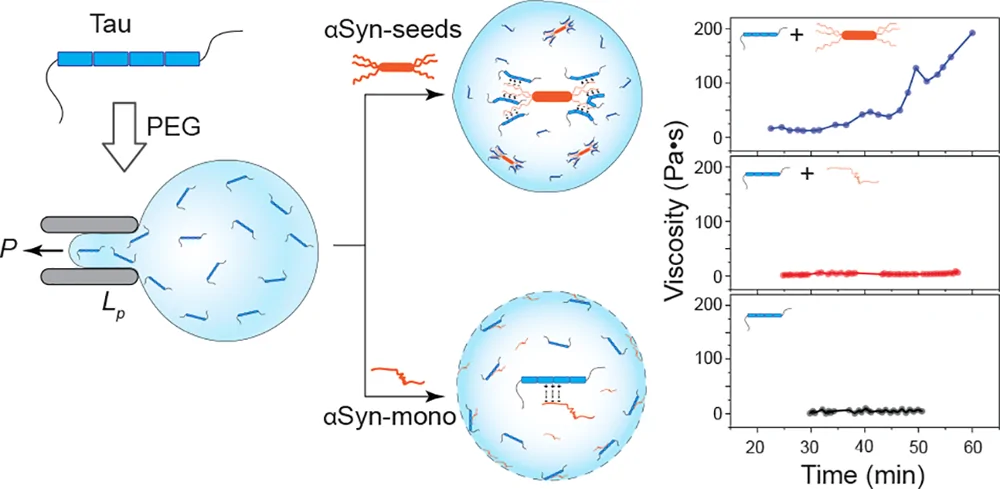

Beyond its canonical functions in microtubule assembly and stabilization, axonal transport, and neuronal signaling^1,2^ Tau can misfold and aggregate under pathological conditions, forming paired helical filaments (PHFs) and neurofibrillary tangles (NFTs), a defining histopathological hallmark of Alzheimer’s disease and related tauopathies.^1,3–5^ Recently, Tau has been shown to undergo phase separation under physiologically relevant conditions in vitro and in cells, forming liquid-like condensates in the presence of cofactors, such as RNA, heparin, and crowding agents like polyethylene glycol (PEG).^6–11^ In addition to homotypic assembly, Tau also exhibits heterotypic complex coacervation with proteins such as αSyn.^12–17^

The full-length Tau 2N4R (441 residues) is an intrinsically disordered protein (IDP) containing 2 N-terminal inserts and 4 microtubule-binding repeats that contribute to its multivalent interactions (Figure S1A).^6,7,10^ αSyn is a 140-residue IDP composed of an amphipathic N-terminal membrane-binding region, a hydrophobic central domain that drives aggregation, and an acidic C-terminal tail (Figure S1B). Aggregated αSyn species form amyloid fibrils that constitute a pathological hallmark of synucleinopathies.

In Alzheimer’s disease, αSyn copathology has been shown to drive Tau accumulation,^18^ highlighting a functional interplay between these two proteins. The crosstalk between αSyn and Tau has been marked by their colocalization and co-occurrence in human brain tissues.^19–21^ Furthermore, immunohistochemical and biochemical studies involving seeding experiments have suggested that each protein can influence the fibrillation of the other.^17,22–25^ However, the molecular basis of how αSyn promotes the accumulation and disease transition of Tau remains unclear.

Recent studies have highlighted the role of biomolecular condensates as dynamic, membrane-less entities that can organize cellular biochemical reactions.^26,27^ Liquid condensates formed by aggregation prone proteins, such as Tau, have been shown to undergo maturation to a solidified state, leading to a proposed role of condensates as intermediates in pathological transitions.^28,29^ Recent evidence also suggests that solidified condensates can kinetically prevent the formation of fibrillar aggregates.^30–35^ Furthermore, the condensate interface has been shown to mediate fibril formation.^32,34,36^

While αSyn is known to partition into Tau condensates,^13,15^ a critical gap persists in determining how distinct αSyn species such as monomers or fibril seeds (seeds) influence the condensates’ material states. The initial transition of liquid condensates into potentially pathological solids corresponds to a rise in steady-state viscosity, while changes to their interface typically perturb interfacial tension. Therefore, quantifications of condensate viscosity and interfacial tension are essential to dissect the mechanistic underpinnings of condensate-related pathologies. Recently, we developed the micropipette aspiration (MPA) technique, a direct and label-free method for simultaneous quantifications of the viscosity and interfacial tension of condensates.^37–40^

In this study, we quantified Tau condensates using MPA and systematically examined how αSyn monomers and seeds modulate their material properties. We focused on the steady-state deformation of condensates and neglected potential short-time scale non-Newtonian behavior.^36,41,42^ We first constructed the phase diagram of purified Tau 2N4R (Figure S1C) in the presence of PEG-8000 (Figures 1A, S1D). To minimize experimental variations, we selected a condition deep in the phase-separated regime (20 μM Tau and 10% PEG-8000) for subsequent condensate material characterization.

**Figure 1:**
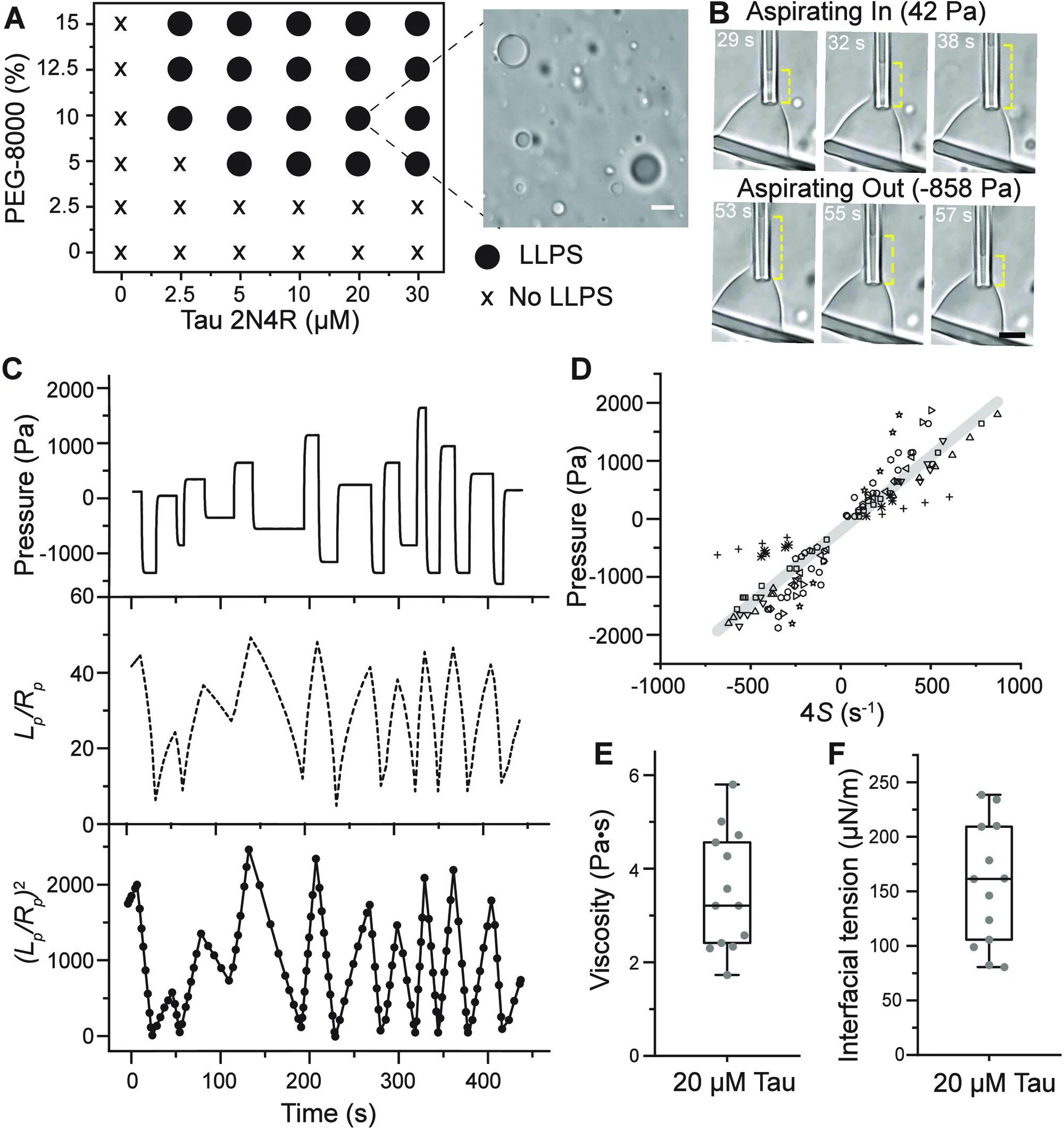
Material properties of Tau condensates. (A) Phase diagram illustrating the formation of Tau condensate with PEG-8000, scale bar 5 μm. (B) Transmitted light images showing changes in the aspiration length (*L*_p_) during MPA, scale bar 10 μm. Yellow dash lines trace *L*_p_. (C) *P*_asp_ (top) and corresponding *L*_p_ (middle; normalized by pipet radius *R*_p_) over time. Calculation of *S* = d­(*L*_p_/*R*_p_)^2^/d*t* under each pressure step (bottom). (D) *P*_asp_ vs 4S for 13 independent condensates. Gray line is a linear fit to all data. (E) Viscosity and (F) interfacial tension of condensates formed by 20 μM Tau in 10% PEG-8000, 25 mM HEPES, 150 mM NaCl, pH 7.4.

Under the time scale of MPA, Tau condensates behave as a Newtonian liquid (Figure 1B, 1C), with a well-defined effective shear rate *S* that changes linearly with the aspiration pressure *P*_asp_ (Figure 1D, eq S1). The viscosity of Tau condensates was 3.5 ± 1.2 Pa·s (mean ± s.d.; same below) and the interfacial tension was 156 ± 55 μN/m (Figure 1E, 1F), in qualitative agreement with condensate fusion-based estimate of capillary velocity (20 ± 3 μm/s; Figure S1E).^43^ We noticed that impurities in recombinant Tau samples can reduce the condensate’s viscosity and interfacial tension (Figure S1F, S1G). In this study, we used the highest purity fractions of Tau from each preparation.

We next examined the effect of αSyn on preformed Tau condensates. Confocal fluorescence microscopy was used to visualize the partitioning of labeled Tau-AF488 and αSyn-AF647 into condensates. Enrichment was quantified by an apparent partition coefficient (*PC*_app_), defined as the ratio of the protein fluorescence in the dense phase to that in the dilute phase. Fluorescent labeling was only used for partitioning measurements, and all MPA experiments were performed with unlabeled proteins to avoid photoartifacts.^44^

Monomeric αSyn partitioned strongly into Tau condensates (Figure 2A, Figure S2A), with a *PC*_app_ decreasing from ∼ 120 to ∼ 45 as total αSyn monomers increased from 5 to 200 μM, consistent with noncooperative recruitment of αSyn into Tau condensates. Notably, under this condition, 200 μM αSyn monomers alone do not form condensates.^45^ The concentration of αSyn monomers in the dense phase is ∼ 5 mM (Figure S2B, S2C), which could have a prominent impact on the material properties of Tau condensates. Using MPA, we found that while the interfacial tension of Tau condensates decreased 3-fold upon the addition of monomeric αSyn (Figure 2C), the viscosity of Tau condensates remained surprisingly stable (Figure 2B, 2D), despite the strong apparent partitioning of αSyn monomers in the condensates. Interestingly, the partitioning of αSyn monomers also did not significantly affect the phase separation of Tau (Figure S2D–S2F).

**Figure 2:**
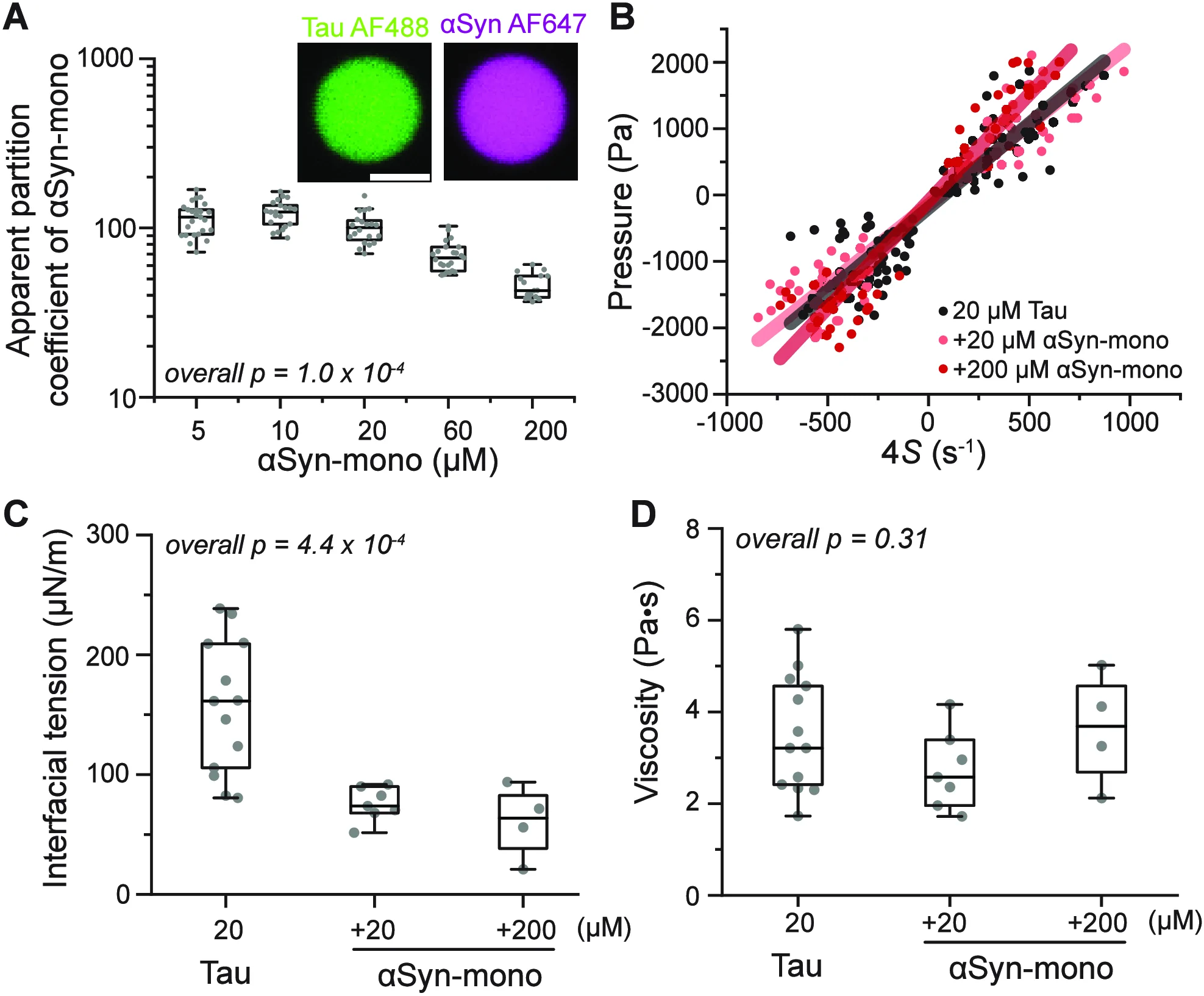
Effect of αSyn monomers on the material properties of Tau condensates. (A) Apparent partition coefficient of αSyn monomers into Tau condensates where Tau is labeled with Tau-AF488, and αSyn with αSyn-AF647. Inset shows representative fluorescence images of the condensate, scale bar 2 μm. (B) *P*_asp_ vs 4*S* plots of Tau condensates in the presence of αSyn monomer. (C) Interfacial tension and (D) viscosity of Tau condensates with increasing concentrations of αSyn monomers.

We next evaluated the effect of αSyn seeds on Tau condensates. In contrast to the intrinsically disordered monomer, αSyn seeds derived from preformed fibrils adopt β-sheet rich cores with flanking disordered termini.^46–48^ The αSyn seeds used here have an average length of 50 nm and average height of 5 nm (Figure S3). Like monomers, αSyn seeds strongly partitioned into Tau condensates, with *PC*_app_∼100 (Figure 3A). Notably, seeds showed heterogeneous accumulation in condensates at 5 μM monomer equivalent concentration (Figure 3B). In contrast to monomers, αSyn seeds substantially increased the viscosity of Tau condensates (Figure 3C, 3D). Even at 1 μM, seeds increased the viscosity of Tau condensates by ∼ 5-fold. Increasing the seeds concentrations to 5 μM resulted in a ∼ 30-fold increase in the average condensate viscosity, while the interfacial tension of condensates was not measurably affected. Importantly, these effects of seeds occurred at αSyn concentrations (≤5 μM) far lower than those used for monomers (up to 200 μM).

**Figure 3:**
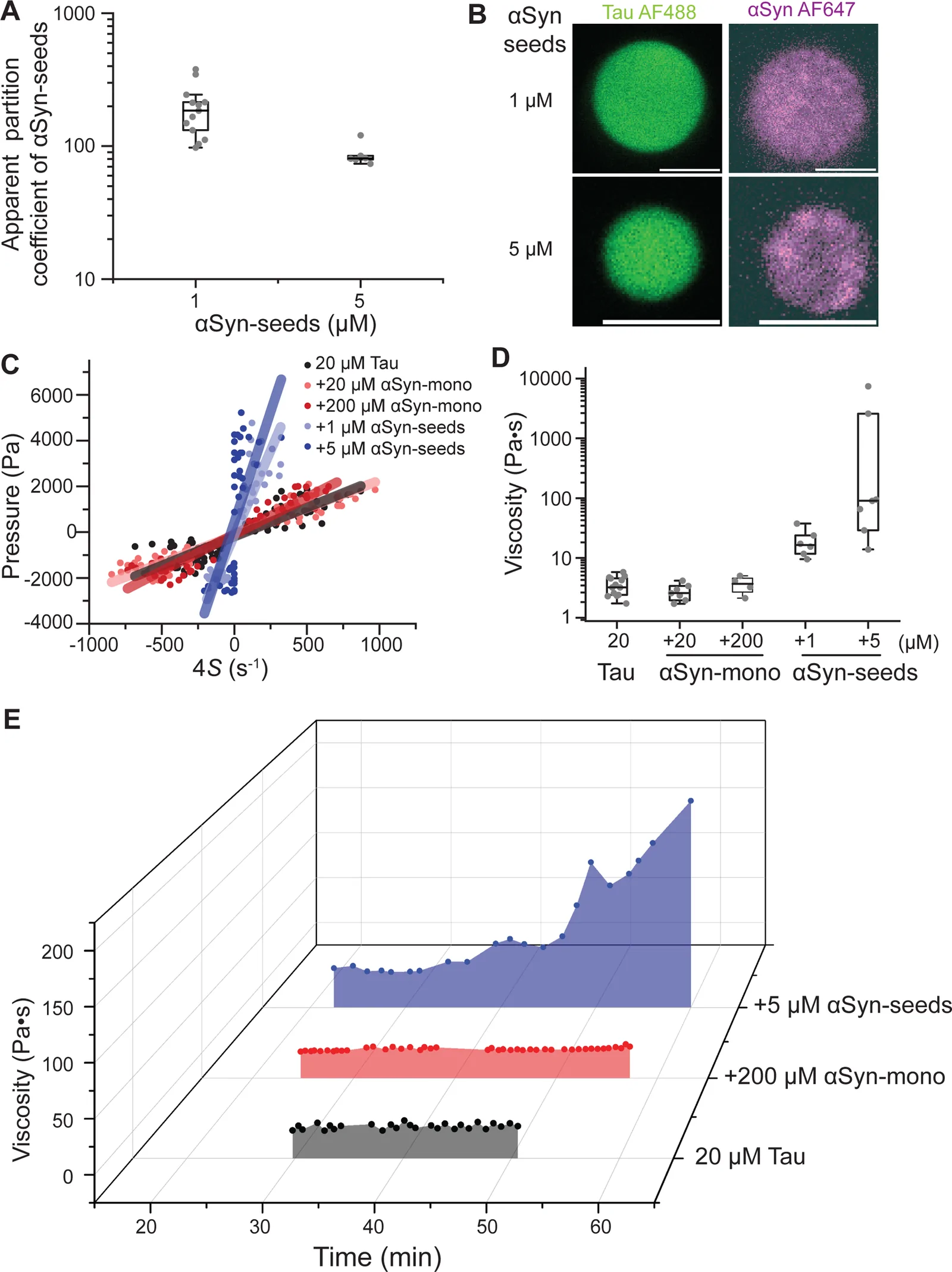
αSyn seeds increase the viscosity of Tau condensates. (A) Partitioning and (B) representative images of αSyn seeds in Tau condensates. Tau is labeled with Tau-AF488, and seeds were made from αSyn monomers containing αSyn-AF647, scale bars 5 μm. (C) *P*_asp_ vs 4*S* plot and (D) viscosity of Tau condensates with αSyn monomers or seeds. (E) 3D plot showing the time-dependence of Tau condensate viscosity without αSyn (black), with 200 μM αSyn monomers (red), or with 5 μM αSyn seeds (blue).

Tracking Tau condensates over 1 h, we observed that αSyn seeds, but not monomers, led to a continuous increase in the measured viscosity (Figure 3E), indicating an apparent maturation of condensates. Despite a significant increase in viscosity, condensates containing αSyn seeds retained near-Newtonian fluid behavior throughout the 1-h MPA tracking window (Figures S4, S5). Nevertheless, we anticipate that substantial elasticity will emerge as the condensates continue to solidify over longer time scales. To determine whether the viscosity increase was associated with fibril formation in condensates, we performed Thioflavin T (ThT) assays on Tau condensates supplemented with αSyn seeds. Consistent with previous studies,^49–53^ αSyn seeds did not induce Tau fibrillation within 24 h, unlike seeded fibrillation of αSyn monomers (Figure S6A). Individual Tau condensates exhibited a basal ThT fluorescence signal that did not change upon incubation with αSyn seeds^54^ (Figure S6B). These results suggest that the increase in the viscosity of Tau condensates (within 1 h of adding αSyn seeds) is not due to fibril formation within the condensates. Instead, it likely reflects the onset of amorphous solid aggregate or gel formation mediated via multivalent interactions (discussed below).^33,55,41,53^

Tau and αSyn are known to interact electrostatically, wherein positively charged proline-rich domains of Tau interact with the negatively charged C-terminal region of αSyn.^17,13,15,16^ At physiological pH, Tau carries a net positive charge (+2) while αSyn monomers are substantially anionic (−9 charge), providing a basis for heterotypic association.^53,17,13^ However, these interactions are transient and do not significantly alter the effective size of diffusing species in fluorescence correlation spectroscopy (FCS) measurements,^53^ in which unlabeled αSyn monomers were added to fluorescently labeled Tau samples. This is consistent with our observations that, despite efficient condensate partitioning, monomeric αSyn alters neither the phase separation of Tau (Figure S2) nor the viscosity of Tau condensates (Figure 2D). Within the framework of dominance analysis,^56^ αSyn monomers can be classified as a nondominant client that does not impact the existing internal network formed by the Tau scaffold. This behavior contrasts with prior observations in Synapsin condensates, where the partitioning of monomeric αSyn significantly increases condensate viscosity and reduces the apparent concentration of Synapsin in the dilute phase.^39^

We also observe that αSyn monomers moderately reduce the interfacial tension of Tau condensates (Figure 2C). While all interfacial tension values reported here fall within the narrow range expected for biomolecular condensates (∼100 μN/m),^36,42^ the effect of αSyn aligns with theoretical predictions that stoichiometric charge mismatch^57^ and protein length asymmetry^58^ (i.e., entropic charge effect) can lower the interfacial tension of heterotypic condensates.

In contrast to its monomeric form, αSyn seeds substantially increase the viscosity of Tau condensates (Figure 3). The viscosity increased within 1 h after mixing αSyn seeds with Tau, far proceed the fibrillation of Tau under our experimental conditions (Figure S6). Interestingly, preformed fibril seeds or oligomers of αSyn are known to further delay the fibrillation of Tau,^52,53^ indicating that αSyn seeds can form stable interactions with Tau which serve as a kinetic trap to prevent Tau fibrillation. Fibrillar αSyn seeds contain a structured amyloid core spanning approximately residues L38–K97,^46–48^ flanked by disordered N- and C- termini that form a dynamic “fuzzy coat”.^59^ While the core provides structural stability, the fuzzy coat mediates key functions,^60,61^ including monomer recruitment^62–64^ and interactions with other proteins.^65–67^ In particular, the highly negatively charged C-terminus remains solvent-exposed^46,68^ along the fibril surface, creating a multivalent binding platform for positively charged biomolecules such as Tau. Consistently, a recent FCS study under nonphase-separating conditions demonstrates that αSyn seeds, but not αSyn monomers, can bind multiple Tau monomers.^53^ We hypothesize that these multivalent interactions, when occurring within condensates, strengthen the macromolecular scaffold and drive a pronounced increase in condensate viscosity. This multivalency-mediated viscosity increase could shed light on the behavior of a broad spectrum of condensates.^69–71^ Additionally, the stronger interaction between Tau and αSyn seeds may induce conformational changes in Tau that expose aggregation domains in the microtubule-binding region.^72,73^ These hydrophobic domains can lead to stronger intermolecular interactions and higher condensate viscosity. However, directly probing molecular interaction strength, valence, or conformation inside condensates requires further technical development and investigation.

Together, our findings demonstrate how distinct αSyn species differentially reprogram the material properties of Tau condensates. Particularly, αSyn seeds lead to a significant increase in the viscosity of Tau condensates, a transition beyond the capacity of αSyn monomers. These observations begin to address a critical gap in our understanding of neurodegeneration by providing a physicochemical framework for how Tau−αSyn crosstalk in neurodegenerative diseases may occur through modulations of condensate material properties.

## Supplementary Material



## Notes

Materials and methods, supplementary figures (Figures S1–S6) and corresponding captions. (PDF)

## †

Campus Straubing for Biotechnology and Sustainability, Technical University of Munich, Schulgasse 16, 94315 Straubing, Germany
