# Supplementary material for: Differential Effects of α‑Synuclein Monomers and Seeds on the Material Properties of Tau Condensates
Source: J Am Chem Soc. 2026 Sep 23;148(39):41717–23. doi: 10.1021/jacs.6c07726 (PMC13651524; doi:10.1021/jacs.6c07726)
Supplement: Supplementary file 1 [file ja6c07726_si_001.pdf]

Supporting Information

Differential effects of α-Synuclein monomers and seeds on the material properties of Tau condensates

Bineet Sharma[†], Jinying Wang[‡], Priscilla Chinchilla Retana[‡], Jean Baum[*], Zheng Shi[*]

Department of Chemistry and Chemical Biology, Rutgers, The State University of New Jersey, Piscataway, New Jersey, USA

[‡]Both authors contributed equally to this work.

*Corresponding authors: Zheng Shi (zheng.shi@rutgers.edu), Jean Baum (baum@chem.rutgers.edu)

The supporting information includes:

Methods and Materials

Supplementary Figure captions

Supplementary Figure S1 to S6

Methods and Materials

Recombinant Tau 2N4R expression and purification

The plasmid pET-29b(+) encoding full-length wild-type human Tau (2N4R) was transformed into *Escherichia coli* BL21(DE3) strain and was plated on Luria Broth (LB) agar supplemented with 50 μg/mL kanamycin and incubated overnight at 37°C. A single colony was then inoculated into a starter culture with 50 mL of LB broth containing 50 μg/mL kanamycin and grown overnight at 37°C with shaking at 180 rpm (Scientific Innova 44 Incubator Shaker, New Brunswick, NJ). 1-2 volume % of the starter culture was then scaled up to 2 L culture and grown again at 37°C at 180 rpm in the presence of kanamycin until OD at 600 nm reaches between 0.5-0.6. For Tau 2N4R expression, *E. coli* culture was induced by 0.5 mM IPTG for 2 hours at 37 °C. Cells with over expressing Tau were collected at 11,500 × g for 15min at 4 °C. The pellet was resuspended in lysis buffer containing 20 mM MES pH 6.8, NaCl 500 mM, EDTA 1 mM, $MgCl_2$ 0.2 mM, DTT 5 mM with protease inhibitor cocktail tablet. A homogeneous pellet in lysis buffer was then sonicated on ice at 30% amplitude with 10 s on and 20 s off for 5 minutes (Sonicator Dismembrator Model 500, Fisher Scientific, MA). Afterwards, the cell lysate was heated over boiling water for 25 minutes and then cooled on ice and centrifuged at 125,000 × g at 4 °C for 50 min using Optima MAX-TL ultracentrifuge. The supernatant was dialyzed with 2 L dialysis buffer (MES 20 mM pH 6.8, NaCl 50 mM, EDTA 1 mM, $MgCl_2$ 2 mM, DTT 2 mM) in a 3.5 kDa snakeskin (Thermo) overnight (~16-18 hours). Tau 2N4R was purified in two steps, one with 2 × 5 mL cationic exchange column (HiTrap SPFF, Cytiva, MA, USA) and other with size exclusion chromatography. Cation exchange column was equilibrated with equilibration buffer (MES 20 mM pH 6.8, NaCl 50 mM, EDTA 1mM, $MgCl_2$ 2 mM, DTT 2 mM before sample injection (dialyzed). Tau containing lysate after dialysis was filtered through a 0.22 μm PVDF filter and injected into the column at a flow rate of 1 mL/min. Tau protein was eluted gradually between 0 – 60% of elution buffer (MES 20 mM pH 6.8, NaCl 1 M, EDTA 1 mM, $MgCl_2$ 2 mM, DTT 2 mM). The fractions containing expected MW tau (confirmed by SDS-PAGE) were pooled and injected again into a size exclusion column (Superdex 75 10 300 GL) with a flow rate of 0.5 mL/min using equilibration buffer. Fractions containing tau analyzed by SDS-PAGE were then buffer exchanged to a desired buffer and stored at -80 °C. Tau concentration was measured using NanoDrop using MW 45849 and extinction coefficient of 7575 $M^{-1}cm^{-1}$.

Recombinant α-Synuclein expression and purification

Expression of non-acetylated human αSyn occurred via transformation into *E. coli* BL21(DE3) cells (New England Biolabs, MA) and plated onto agar plates containing the antibiotic ampicillin (50 μg/mL). 50 mL starter cultures were inoculated using one colony each and grown in LB media at 37°C, 160 rpm overnight (Scientific Innova 44 Incubator Shaker, New Brunswick, NJ). 1L LB cultures were inoculated using 10 mL starter cultures and were allowed to grow at 37°C, 180 rpm until an O.D of 0.6 was reached. At

this point, the cultures were induced with 1 mM IPTG and grown at 20°C, 180 rpm for 16-18 hours. After overexpression, cultures were pelleted down and stored at -80 °C until use.

Non-acetylated human αSyn was purified by resuspending the bacterial pellets in 10 mM PBS (pH 7.4). The protein solutions were lysed via sonication (Sonicator Dismembrator Model 500, Fisher Scientific, MA) at 30% amplitude for 8 cycles of 15 seconds on 30 seconds off. Solutions were kept on ice to prevent degradation. Following lysis, samples were boiled for 20 minutes and centrifuged at 20,000 rpm for 45 minutes (Avanti J-26S XPI Centrifuge, Beckman Coulter, CA) to remove cellular debris. The supernatants were collected and treated with streptomycin sulfate (10 mg/mL) to remove nucleic acids. The solutions were mixed at 4°C for 20 minutes and centrifuged as previously described. The protein was precipitated by add-ing ammonium sulfate (0.361 g/mL) and mixed at 4°C for 1 hour after which the solutions were centri-fuged once more to collect the final protein pellets. The pellets were resuspended in Buffer A (32 mM Tris, pH 7.8) and prepared for fast protein liquid chromatography (FPLC) by filtration using a 0.22 μm syringe filter. The filtered protein solutions were injected onto a HiTrap Q HP anion exchange column (Cytiva AKTA, MA) and the purified protein was eluted at 50% Buffer B (32 mM Tris, 500 mM NaCl, pH 7.8) using an AKTA Pure FPLC (Cytiva AKTA, MA). The purified protein was flash frozen using LN2 and stored at -80 °C until use.

α-Synuclein fibril seeds preparation

Flash-frozen αSyn monomers was thawed on ice, and large aggregates were removed using a 50 kDa centrifugal filter (Millipore Sigma, St. Louis, MO). The protein was then concentrated into 10 mM PBS (pH 7.4) using a 3 kDa centrifugal filter (Millipore Sigma) to a final concentration of 300–500 μM. 500 μL of the concentrated protein was aliquoted into a 1.5 mL microcentrifuge tube and incubated in an orbital thermomixer (Thermomixer C, Eppendorf, MA) at 37 °C with shaking at 1000 rpm for 7 days. Fibrils were collected by centrifugation at 15,000 rpm for 2 hours (Microcentrifuge 5430, Eppendorf, MA). The resulting pellets were washed via multiple rounds of resuspension in 10 mM PBS (pH 7.4) followed by centrifugation at 15,000 rpm for 2 hours to remove residual soluble and non-fibrillar species. Fibril pellets were stored at room temperature until use. Fibril seeds were prepared by sonicating 1 mg/mL of fibrillar protein (15 seconds on, 15 seconds off, 30% amplitude) for 3 cycles on ice. AFM samples were prepared by diluting seeds at a 1:10 concentration. The concentrations of fibrils and seeds were determined by dissociating in guanidinium and performing a bicinchoninic acid assay. The concentration is then reported in monomer equivalents.

Air AFM imaging

For the air AFM imaging sample preparation, 50 μL of fibril seeds were placed onto freshly cleaved mica and allowed to bind to the surface for approximately 5 minutes. After, the mica surface was washed with 2

mL of Milli-Q water, and the sample was dried at room temperature for 2 hours in a laminar flow hood. After drying, the sample was placed into the AFM for imaging (Cypher ES AFM, Asylum Re-search, Oxford Instruments, United Kingdom). Imaging was performed at 25°C with AC240 tips (nominal resonance frequency of 70 kHz, nominal spring constant of 2 N/m).

Tau phase separation in presence of a crowding agent

The phase separation of Tau was induced by supplementing polyethylene glycol (PEG-8000, Thermo Fisher Scientific) as a crowding agent in HEPES buffer (25 mM HEPES, 150 mM NaCl, pH 7.4) at room temperature. As shown in Figure 1, Tau with varying final concentration of PEG-8000 were mixed in a low binding tubes by slow pipetting and observed under microscope using homemade observation chamber fabricated by sandwiching two cover glasses with a layer of vacuum grease in between. Tau condensates were observed using Nikon Ti2-A inverted fluorescence microscope with 60 x objective unless otherwise mentioned.

Fluorescent labelling of Tau and α-Synuclein

The presence of two native cysteines in Tau at 291 and 322 were site-specifically labeled by maleimide-thiol reaction strategy using AlexaFluor488 C5-maleimide following manufacturer's protocol (Thermo Fisher Scientific). Briefly, purified Tau was buffer exchanged into labelling buffer (25mM HEPES, 150mM NaCl, pH 7.4) using Zeba™ Spin Desalting columns (Thermo Fisher Scientific). Tau and AF488 were mixed in the molar ratio of 1:1000 and incubated overnight at 4 °C. The excess dye was removed using Zeba™ spin columns.

Similarly, for αSyn, single cysteine mutation at A140C was employed for site-specific labeling with AlexaFluor647 C2-maleimide in the molar ratio of 1:1000 and incubated overnight at 4 °C. The excess dye was removed by Zeba™ spin columns and concentration of labelled proteins were measured using NanoDrop with absorbance correction based on manufacturer's protocol.

Partitioning of α-Synuclein monomers & seeds into Tau condensates

Tau condensates were freshly prepared by mixing 20 μM Tau (15 μM unlabeled Tau: 5 μM labeled Tau-AF488) with 10% PEG-8000 in HEPES buffer and then supplemented with varying concentration of αSyn monomers (x μM unlabeled αSyn: 5 μM labeled αSyn-A140C-AF647). The protein mixed solution was transferred to a glass slide precoated with 5% Pluronic acid (F-127, Sigma Aldrich). An observation chamber was created by employing a silicone spacer of 0.5 mm and condensates were observed under confocal microscopy, Zeiss Axio Observer 7 inverted microscope equipped with an LSM900 laser scanning module. Similarly, partitioning of labelled preformed α-Synuclein seeds was conducted under identical conditions. All images were analyzed using Fiji ImageJ.

Thioflavin T (ThT) assays

For microscopy imaging of ThT, stock solution of Thioflavin T (ThT; Thermo Scientific Chemicals and Acros Organics, Pittsburgh, PA, CAS 2390-54-7) was prepared in HEPES buffer (25 mM HEPES, 150 mM NaCl, pH 7.4). For this assay, unlabeled preformed αSyn fibril seeds, Tau, and ThT were combined in desired concentrations in the presence or the absence of PEG-8000 (Figure S3D). ThT was added last to initiate the assay with a final concentration of 20 μM in all samples. ThT assay was performed under four conditions: (i) αSyn seeds + ThT, (ii) Tau + αSyn seeds + ThT, (iii) Tau + PEG-8000 + ThT, and (iv) Tau + αSyn seeds + PEG-8000 + ThT. Samples were imaged using Nikon Ti2-A inverted fluorescence microscope at room temperature for 2 h.

For bulk ThT assay, tau monomer was diluted to a final concentration of 40 μM. A higher concentration of Tau was chosen here to ensure the bulk sample contains sufficient dense phase under phase-separating conditions. When indicated, αSyn fibril seeds were added at a final concentration of 5 μM and PEG-8000 was added at a final concentration of 10%. We also used αSyn monomer at a final concentration of 70 μM as a positive control, as it undergoes robust fibrillization in the presence of αSyn seeds.[1] ThT was added to all samples at a final concentration of 20 μM. Samples were loaded into 96-well plates (Corning, Corning, NY), sealed with Axygen sealing film (Corning, Corning, NY) and incubated under quiescent conditions at 37 °C. ThT fluorescence was monitored using a POLARstar Omega plate reader (BMG LABTECH, Cary, NC). At least four replicates were recorded for each condition. Fluorescence traces were averaged and the standard deviation of these traces is presented in Figure S6A.

Micropipette aspiration (MPA)

In this method, micropipettes were fabricated using a thin-walled glass capillaries TW100-4 (OD = 1.0 mm, ID = 0.7 mm, length = 100 mm) by a precision puller instrument (PUL-1000, World Precision Instruments). Each capillary tube yielded two fine capillaries. The tip of the capillary was then carefully trimmed to form an orifice, typically ranging between 2 to 5 μm in diameter. Following this, the tip was bent to an angle of ~ 60° using a microforge device (DMF1000, World Precision Instruments) to help in positioning during MPA experiments. This finely tuned micropipette was then filled with appropriate buffer solution and attached with external pressure pump (FLUIGENT, ESORT-PCK01). Before each aspiration experiment, zero pressure was carefully measured using milli-Q water in a glass-bottom dish.

MPA experiments were performed on Nikon Ti2-A inverted fluorescence microscope with 60 x objective equipped with motorized stage and two micromanipulators (PatchPro-5000, Scientifica), an optical tweezers (Tweez305, Aresis) and a pressure pump (FLUIGENT, ESORT-PCK01). In the experimental setup, protein condensates were transferred to a glass bottom dish mounted over the microscope. One pipette with fused orifice acted as a holding pipette to immobilize the protein condensate for better aspiration

recording, while another pipette was connected to pressure flow control set up. To minimize sample evaporation, a local humidity environment was created by adding Milli-Q water to the outer region of the glass-bottom dish.

Because large condensates increase the precision of MPA measurements, we used optical tweezers under minimal laser power to gently fuse several micron-sized Tau condensates until they reach over 5 μm in diameter. The optical tweezers only served to position two condensates in contact and not to mechanically induce the fusion. During fusion, the relaxation time of the droplet aspect ratio was analyzed using a stretched-exponential fitting approach.[2,3] Then, the capillary velocity of the condensate is calculated by dividing the geometric mean of the condensate diameters by the relaxation time. Following each fusion event, condensates were allowed to fully relax to a stable spherical shape, typically within 10 s (Figure S1E and S4A). The final fused condensate was then equilibrated for 5 min before aspiration to ensure stable free interfaces and minimize transient effects arising from post-fusion relaxation (Figure S4B). Prior to data acquisition, initial aspiration cycles were performed to equilibrate the condensate with the micropipette, as described previously.[4]

This single condensate was positioned on a sealed holding capillary and aspirated into another ~3 μm glass micropipette under external pressure. Positive pressure was applied to aspirate the condensate into the capillary, called "aspirating-in" while negative pressure pulled it back called "aspirating-out", generating a corresponding change in the aspirated length (Figure 1B). It is important to note that condensate fusion and relaxation processes could in principle influence rheological measurements.

The viscosity and interfacial tension of Tau condensates were determined by MPA using the linear relationship between aspiration pressure (P_{asp}) and effective shear rate (S). Following eq S1, the slope and intercept of P_{asp} vs. S give the viscosity and interfacial tension of the aspirated condensate, respectively.

$$P_{\mathrm{asp}} = 4 \cdot \eta \cdot S - 2\gamma H \qquad \text{(S1)}$$

Where P_{asp} is the aspiration pressure, η is the viscosity of the aspirated condensate, γ is the interfacial tension of the condensate, H is the radius of curvature of the condensate interface inside pipette. S is the effective shear rate defined as $S = \mathrm{d}\,(L_{\mathrm{p}}/R_{\mathrm{p}})^2/\mathrm{dt}$, where L_{p} is the length of the condensate aspirated inside the micropipette and R_{p} is the radius of the micropipette. The equation assumes that the characteristic size of the condensate outside the pipette is much larger than R_{p}. It is important to note that MPA measures the bulk viscosity on the length scale of the micropipette opening, effectively averaging out potential submicron heterogeneities in condensate material properties.

Statistical analysis

Statistical information for all figures is summarized below. In box plots, the central lines denote the median, the boxes span the interquartile range (25 to 75%), and the whiskers extend to 1.5 times the interquartile range.

Figure 1D. Linear regression of aspiration pressure vs. 4S for 13 independently prepared; R^2 = 0.84.

Figure 2B. Linear regression of pressure vs. 4S plots of Tau condensates in the presence of αSyn monomer as measured by MPA; R^2 = 0.91 for Tau + 20 μM αSyn; R^2 = 0.90 for Tau + 200 μM αSyn. (C-D) p values were determined using one-way analysis of variance (ANOVA).

Figure 3C. Linear regression of pressure vs. 4S plot of Tau condensates in the presence of αSyn seeds; R^2 = 0.80 for Tau + 1 μM αSyn-seeds; R^2 = 0.41 for Tau + 5 μM αSyn-seeds.

Supplementary Figure captions

Supplementary Figure S1. Domain architecture, charge distribution of αSyn & Tau, and Tau 2N4R phase diagram.

(A) Schematic representation of Tau 2N4R domains, including N-terminal inserts (N1, N2), proline-rich regions (P1, P2), and microtubule-binding repeat domains (R1–R4). The net charge per residue (NCPR) profile highlights alternating positively (blue) and negatively (red) charged regions along the sequence. (B) Domain organization of αSyn showing the amphipathic N-terminal region, hydrophobic NAC domain, and acidic C-terminus, with the corresponding NCPR profile indicating charge segregation. (C) SDS-PAGE analysis of Tau 2N4R fractions, showing purified ("pure") and less purified ("impure") samples. (D) Bright-field microscopy images showing phase separation of Tau 2N4R as a function of protein concentration (0–30 μM) and PEG-8000 concentration (0–15%). Highlighted image represents 20 μM Tau with 10% PEG-8000 used in this study, scale bar, 10 μm. (E) Time-lapse images showing optical tweezers assisted fusion of Tau condensates. Scale bars: 5 μm. (F) Comparison of bulk viscosity measurements of Tau pure fraction to the impure fraction ($p = 1.8 \times 10^{-4}$). (G) Interfacial tension measurements reveal reduced surface tension in the impure fraction relative to the pure fraction ($p = 0.009$), indicating altered material properties.

Supplementary Figure S2. αSyn partitions into Tau condensates in a concentration-dependent manner.

(A) Confocal fluorescence images of mixed condensates formed by 20 μM Tau (15 μM unlabeled and 5μM AF488-labeled) and increasing concentrations of αSyn (5–200 μM, with fixed 5 μM AF647-labeled αSyn). αSyn efficiently enriches within Tau droplets with increasing concentration, while Tau remains robustly partitioned across conditions. Scale bars, 2 μm. (B, C) Quantification of effective fluorescence intensity of αSyn in the dense (B) and dilute (C) phases as a function of αSyn concentration, showing enhanced partitioning into condensates at higher concentrations. (D, E) Corresponding quantification of Tau fluorescence intensity in the dense (D) and dilute (E) phases. Tau enrichment in the dense phase and depletion from the dilute phase remain relatively stable across increasing αSyn concentrations, indicating minimal perturbation of Tau partitioning. From Figure (B) & (C), fluorescence intensities were converted to concentrations using a linear calibration factor, assuming saturation exists at 200 μmM αSyn, ($C = I_{\text{dense/dil}} / I_{200} \times 200$ μmM), yielding an estimated ~5 mM enrichment of αSyn in the dense phase. Where C = estimated αSyn concentration, $I_{\text{dense/dil}}$ = average fluorescence intensity of dense or dilute phase, I_{200} = average fluorescence intensity at 200 μmM αSyn. (F) Background-corrected dilute-phase fluorescence of Tau condensates (black; labeled with Alexa Fluor 488) or Synapsin condensates (red; labeled with EGFP) vs. the concentration of added αSyn monomer. The Tau data is reproduced from E. The Synapsin data is

reproduced from data associated with Figure S10 of reference[5]. All data are shown as mean ± standard error. Lines are linear fits. Slope of black line = -0.04 ± 0.29 A.U./μM. Slope of red line slope = -7.1 ± 0.8 A.U./μM.

Supplementary Figure S3. Characterization of α-Syn fibril seeds by atomic force microscopy (AFM).
(A) Representative atomic force microscopy images of sonicated αSyn fibril seeds, with an average length of 49.2 ± 23.6 nm (B) and height of 4.74 ± 0.72 nm (C) (mean ± s.d.), scale bar 400 nm.

Supplementary Figure S4. Optical tweezers-assisted fusion dynamics and micropipette aspiration of Tau condensates containing αSyn seeds.
(A) Time-lapse images showing fusion of Tau condensates in the presence of 5 μM αSyn seeds. (B) Representative MPA characterization of Tau condensates on a holding pipette in the presence of 5 μM αSyn seeds. Scale bars: 5 μm in A, 10 μm B.

Supplementary Figure S5. Time-resolved characterization of Tau condensates in presence of αSyn seeds.
(A) Traces of aspiration pressure (top), normalized condensate deformation (middle; L_p: aspiration length, R_p: radius of micropipette), and time-resolved viscosity (bottom) for Tau condensates with 5 μM αSyn seeds. (B) Segmented condensate deformation under each pressure step. The initial deformation (L_0) at the start of each pressure step (t_0) was first subtracted. Then the corrected deformation was normalized by the corresponding aspiration pressure (P) following reference.[6] A capillary pressure (P_γ) of -150 Pa, based on measured condensate interfacial tension, was used to correct the pressure applied to the condensate. Each segmented trace was color-coded (see color bar) by the starting time of its respective pressure step. (C) Traces in B normalized by their corresponding maxima. The response of a purely viscous liquid is represented by a diagonal line. Departure from this diagonal indicates the emergence of elasticity, whereas a purely elastic solid would exhibit a step-function response.

Supplementary Figure S6. Thioflavin T (ThT) assay of Tau under PEG-induced phase separation in the presence of αSyn seeds.
(A) Bulk ThT assay under quiescent condition. Black: Tau condensates without αSyn seeds (40 μM Tau + 10% PEG-8000). Cyan: Tau monomers with αSyn seeds (40 μM Tau + 5 μM αSyn seeds). Blue: Tau condensates with αSyn seeds (40 μM Tau +10% PEG-8000+ 5 μM αSyn seeds), this condition mimics our MPA experiments in Figure 3. Purple: αSyn monomers with αSyn seeds (70 μM αSyn + 5 μM αSyn seeds), this condition serves as a positive control for fibril formation. ThT was added to all samples at a final concentration of 20 μM. At least four replicates were recorded for each condition, error bars represent

standard deviation. (B) Fluorescence images of ThT assays of Tau (20 μM) in the absence and presence of αSyn seeds (5 μM) and PEG-8000 (10%), recorded at t = 0 and 120 min. Under PEG-induced phase separation, Tau condensates exhibit a baseline ThT signal that does not show a significant increase upon addition of αSyn seeds, indicating no substantial enhancement of ThT-positive species within condensates. Fresh αSyn seeds with comparable AFM-based morphological quantifications were used for each experiment.

Figure S1

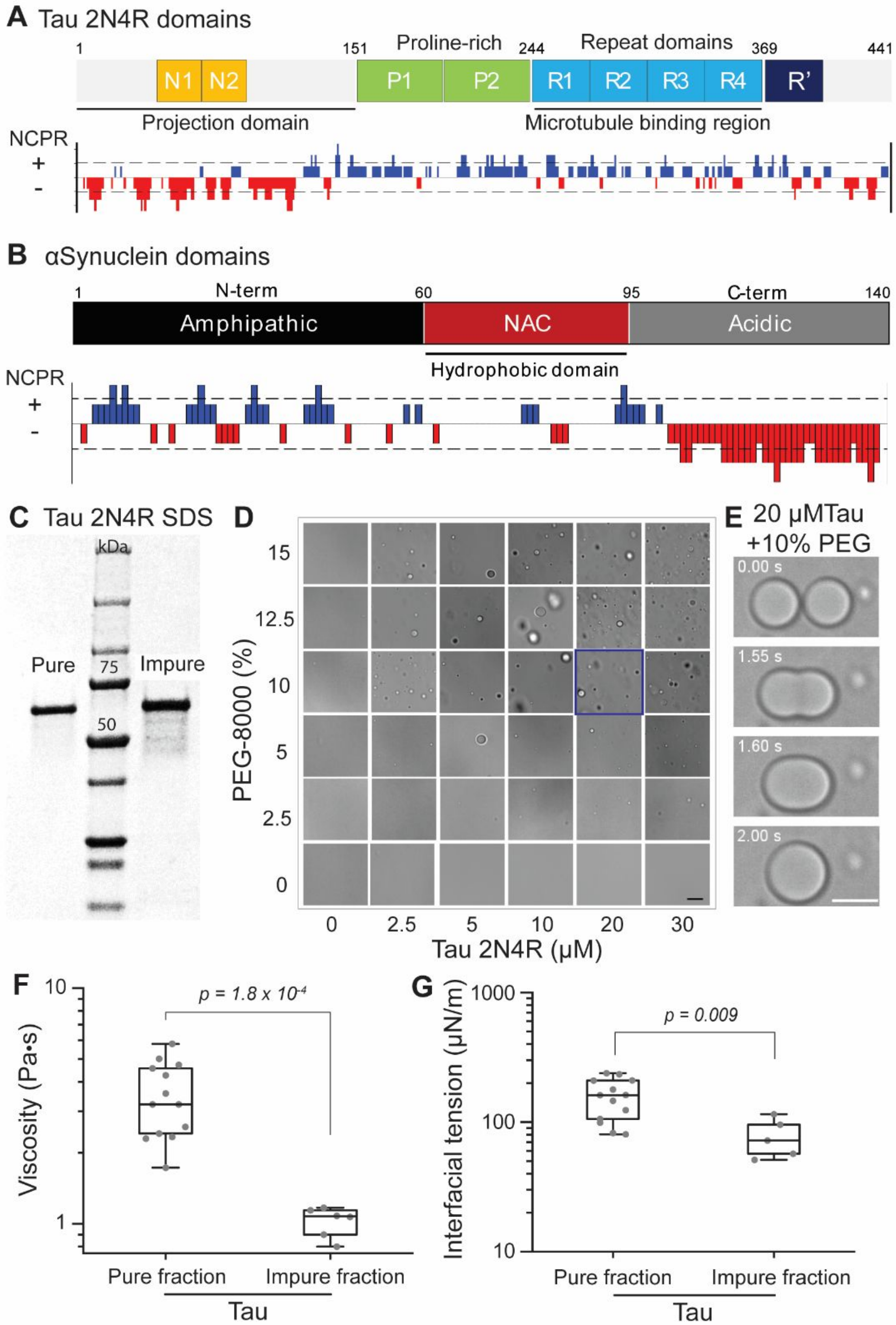
A Tau 2N4R domains
1
151
Proline-rich
244
Repeat domains
369
441
N1 N2
P1
P2
R1
R2
R3
R4
R'
Projection domain
Microtubule binding region
NCPR
+
-
B αSynuclein domains
1
N-term
60
95
C-term
140
Amphipathic
NAC
Acidic
Hydrophobic domain
NCPR
+
-
C Tau 2N4R SDS
kDa
Pure
75
Impure
50
D
PEG-8000 (%)
15
12.5
10
5
2.5
0
0
2.5
5
10
20
30
Tau 2N4R (μM)
E 20 μMTau
+10% PEG
0.00 s
1.55 s
1.60 s
2.00 s
F
Viscosity (Pa•s)
10
1
p = 1.8 x 10-4
Pure fraction
Impure fraction
Tau
G
Interfacial tension (μN/m)
1000
100
10
p = 0.009
Pure fraction
Impure fraction
Tau

Figure S2

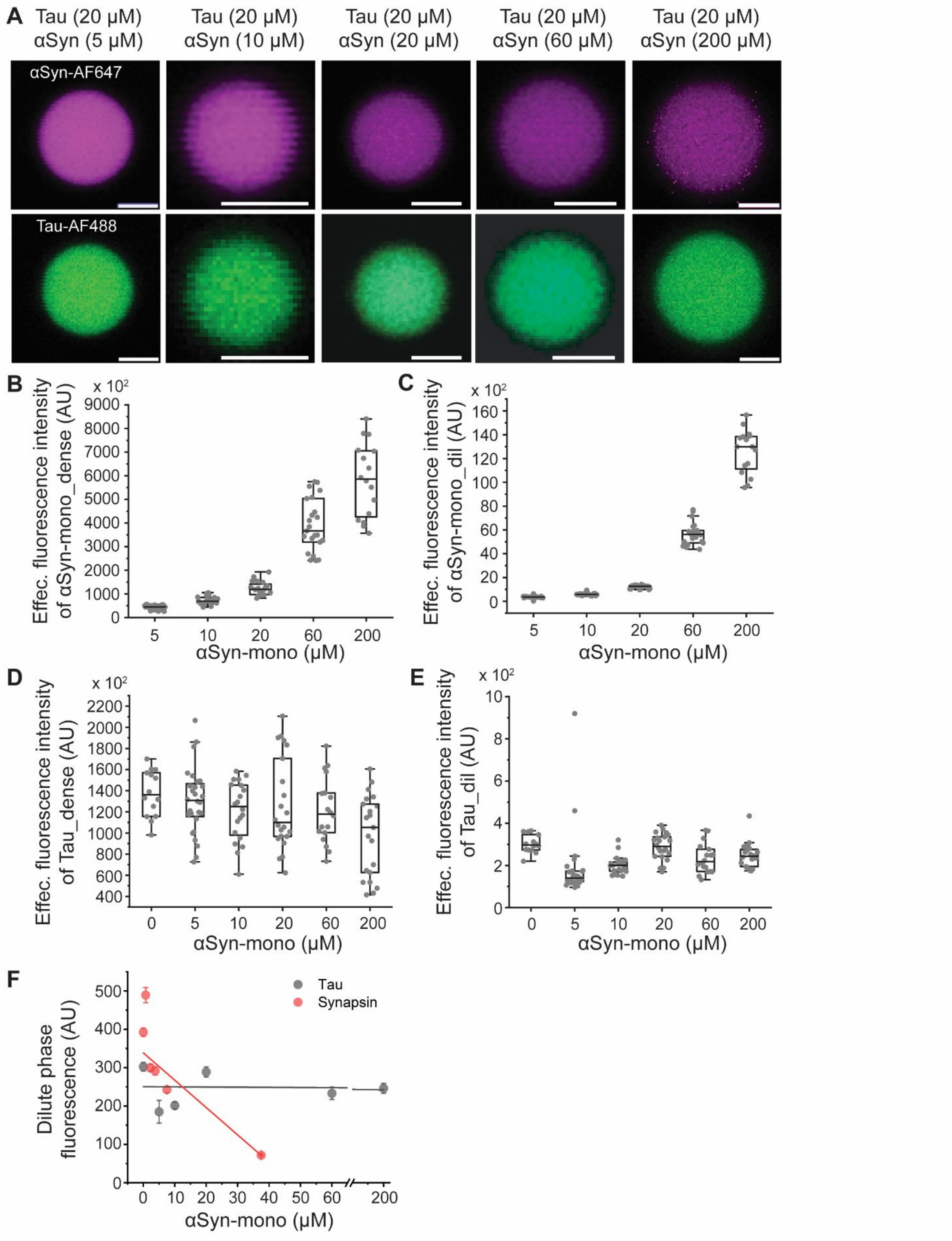
A
Tau (20 μM) αSyn (5 μM)
Tau (20 μM) αSyn (10 μM)
Tau (20 μM) αSyn (20 μM)
Tau (20 μM) αSyn (60 μM)
Tau (20 μM) αSyn (200 μM)
αSyn-AF647
Tau-AF488
B
Effec. fluorescence intensity of αSyn-mono_dense (AU)
x 10²
αSyn-mono (μM)
C
Effec. fluorescence intensity of αSyn-mono_dil (AU)
x 10²
αSyn-mono (μM)
D
Effec. fluorescence intensity of Tau_dense (AU)
x 10²
αSyn-mono (μM)
E
Effec. fluorescence intensity of Tau_dil (AU)
x 10²
αSyn-mono (μM)
F
Dilute phase fluorescence (AU)
Tau
Synapsin
αSyn-mono (μM)

Figure S3

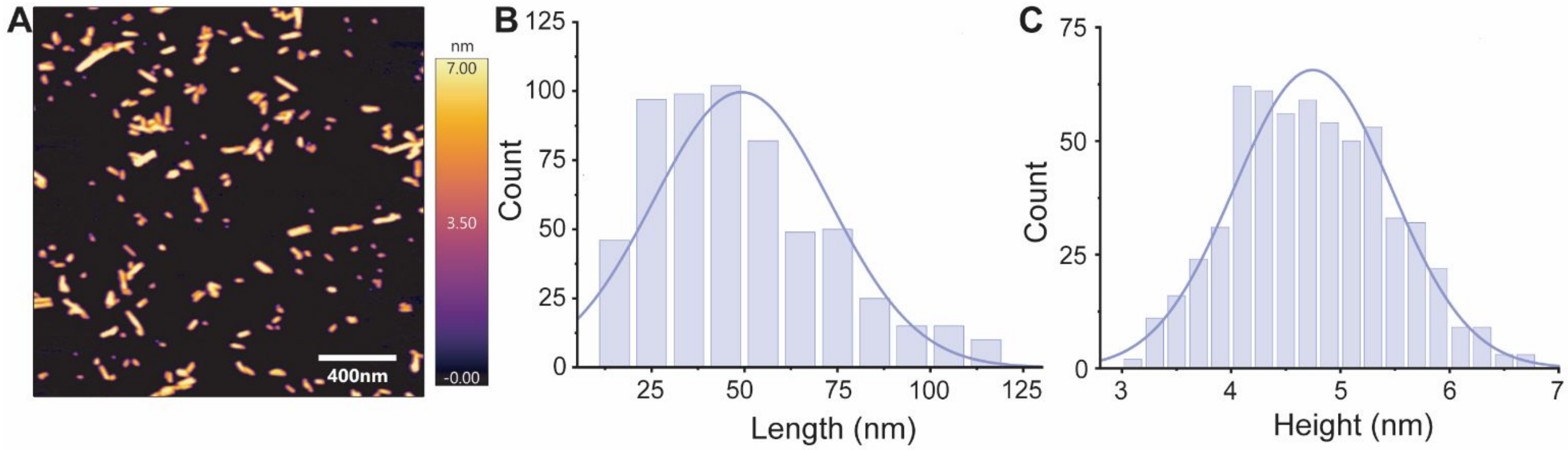

Figure S4

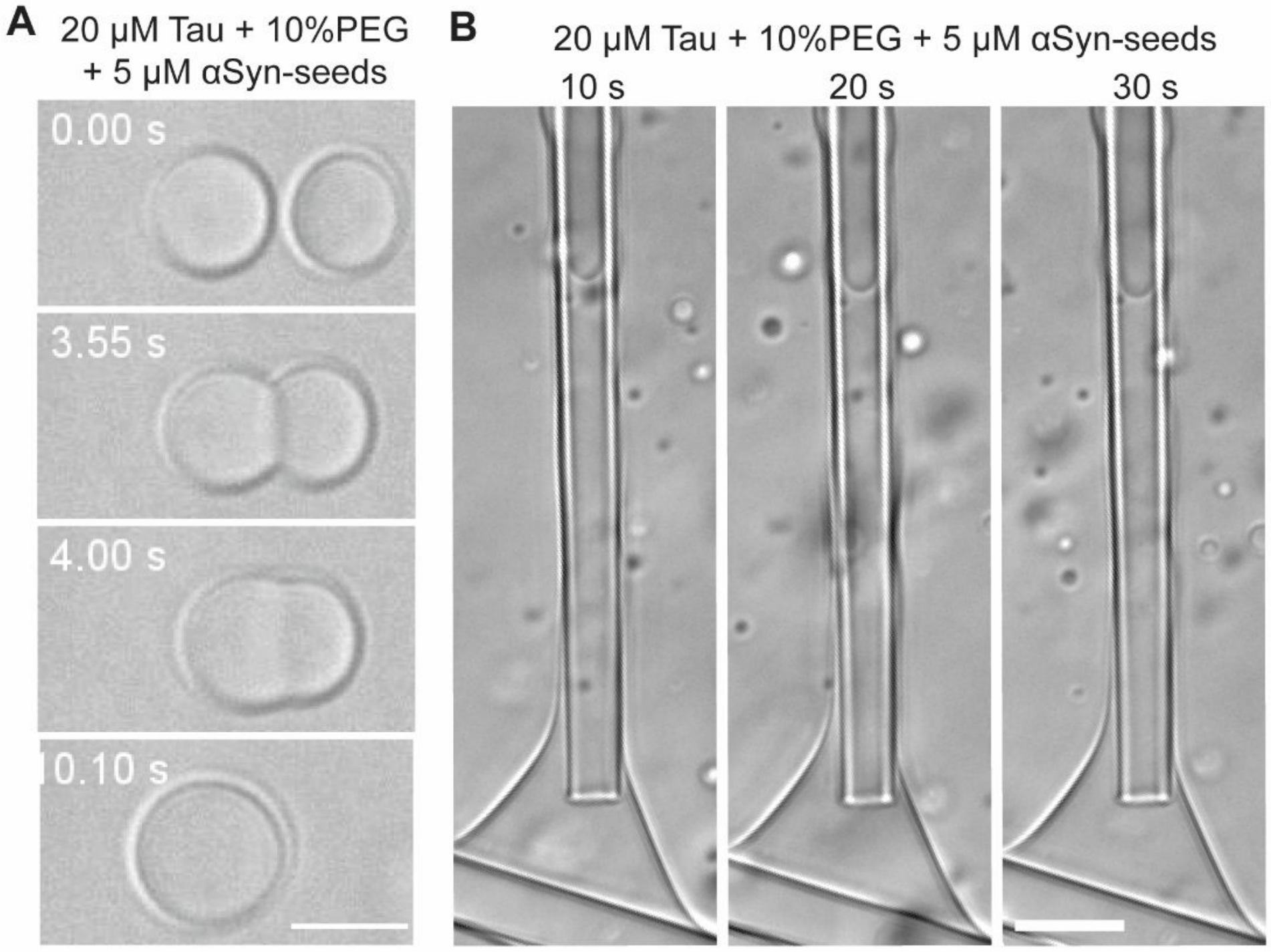
A
20 μM Tau + 10%PEG
+ 5 μM αSyn-seeds
0.00 s
3.55 s
4.00 s
10.10 s
B
20 μM Tau + 10%PEG + 5 μM αSyn-seeds
10 s
20 s
30 s

Figure S5

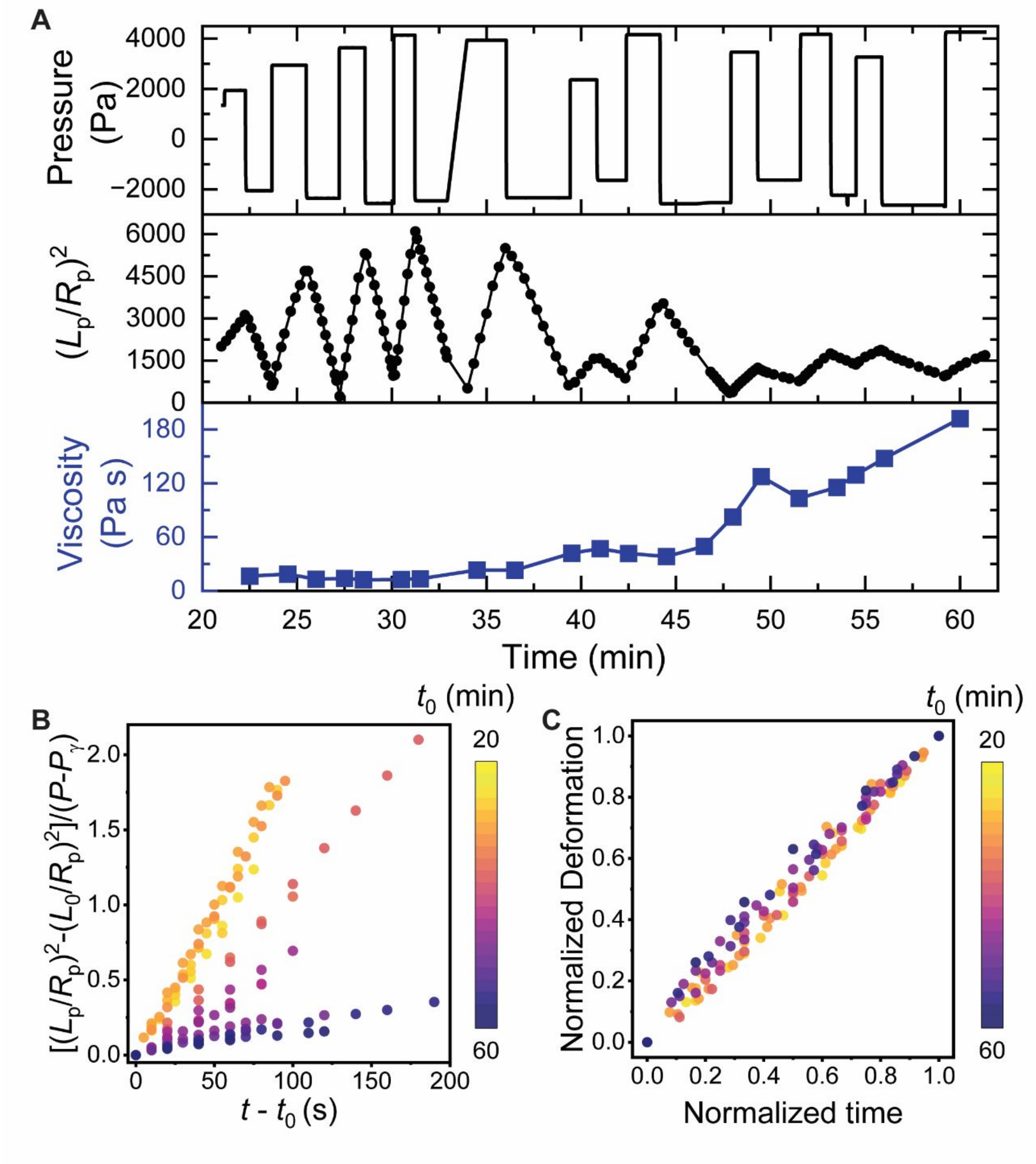

A
Pressure (Pa)
4000
2000
0
−2000
$(L_p/R_p)^2$
6000
4500
3000
1500
0
Viscosity (Pa s)
180
120
60
0
20
25
30
35
40
45
50
55
60
Time (min)
B
$[(L_p/R_p)^2-(L_0/R_p)^2]/(P-P_\gamma)$
2.0
1.5
1.0
0.5
0.0
0
50
100
150
200
$t - t_0$ (s)
t_0 (min)
20
60
C
Normalized Deformation
1.0
0.8
0.6
0.4
0.2
0.0
Normalized time
t_0 (min)
20
60

Figure S6

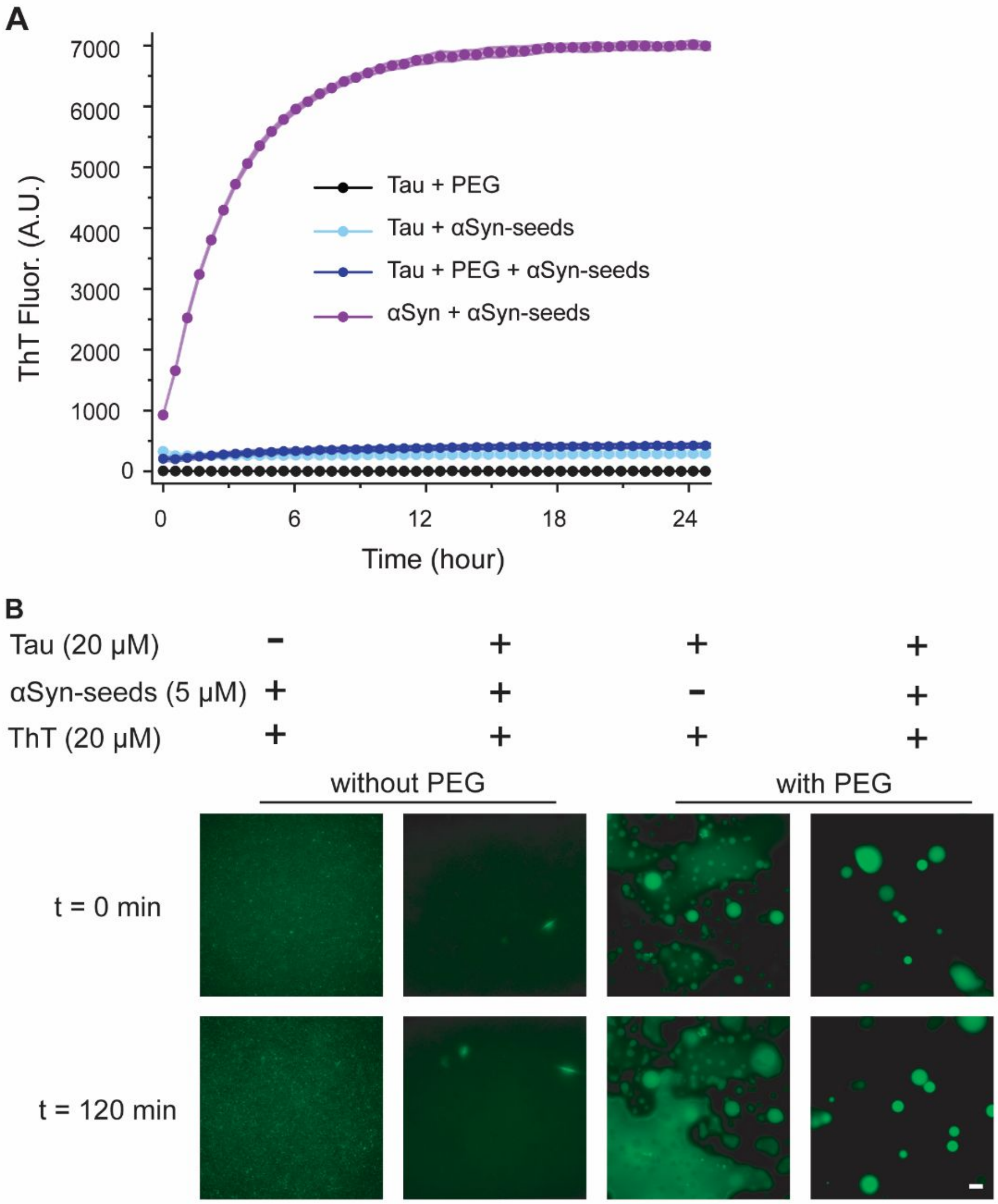
A
7000
6000
5000
4000
3000
2000
1000
0
ThT Fluor. (A.U.)
0
6
12
18
24
Time (hour)
Tau + PEG
Tau + αSyn-seeds
Tau + PEG + αSyn-seeds
αSyn + αSyn-seeds
B
Tau (20 μM) - + + +
αSyn-seeds (5 μM) + + - +
ThT (20 μM) + + + +
without PEG
with PEG
t = 0 min
t = 120 min

References:

(1) Dewison, K. M.; Taylor, A. I. P.; Brockwell, D. J.; Radford, S. E. Mapping the Role of Monomer Conformation in the Amyloid Formation of α-Synuclein Splice Variants. *J. Am. Chem. Soc.* **2025**, *147* (44), 40519–40536. https://doi.org/10.1021/jacs.5c12366.

(2) Favetta, B.; Wang, H.; Shi, Z.; Schuster, B. S. Amphiphilic Protein Surfactants Reduce the Interfacial Tension of Biomolecular Condensates. *Langmuir* **2025**, *41* (35), 23827–23836. https://doi.org/10.1021/acs.langmuir.5c03118.

(3) Naderi, M. M.; Peng, Z.; Zhou, H.-X. Droplet Fusion as a Relaxation Process: Comparison with Shape Recovery of Newtonian and Viscoelastic Droplets. arXiv June 24, 2026. https://doi.org/10.48550/arXiv.2606.26272.

(4) Wang, H.; Kelley, F. M.; Milovanovic, D.; Schuster, B. S.; Shi, Z. Surface Tension and Viscosity of Protein Condensates Quantified by Micropipette Aspiration. *Biophysical Reports* **2021**, *1* (1), 100011. https://doi.org/10.1016/j.bpr.2021.100011.

(5) Wang, H.; Hoffmann, C.; Tromm, J. V.; Su, X.; Elliott, J.; Wang, H.; Deng, M.; McClenaghan, C.; Baum, J.; Pang, Z. P.; Milovanovic, D.; Shi, Z. Live-Cell Quantification Reveals Viscoelastic Regulation of Synapsin Condensates by α-Synuclein. *Science Advances* **2025**, *11* (16), eads7627. https://doi.org/10.1126/sciadv.ads7627.

(6) Roggeveen, J. V.; Wang, H.; Shi, Z.; Stone, H. A. A Calibration-Free Model of Micropipette Aspiration for Measuring Properties of Protein Condensates. *Biophysical Journal* **2024**, *123* (11), 1393–1403. https://doi.org/10.1016/j.bpj.2023.09.018.
